# Dietary Intake Is Not Associated with Body Composition nor with Biochemical Tests but with Psychological Status of Cancer Patients Receiving Chemotherapy

**DOI:** 10.3390/nu15245087

**Published:** 2023-12-13

**Authors:** Hadil S. Subih, Esraa A. Al-Shwaiyat, Nahla Al-Bayyari, Belal S. Obeidat, Fadi Abu-Farsakh, Hiba Bawadi

**Affiliations:** 1 Department of Nutrition and Food Technology, Faculty of Agriculture, Jordan University of Science and Technology, P.O. Box 3030, Irbid 22110, Jordan; esraashweiat@gmail.com; 2 Department of Nutrition and Food Processing, Faculty of Al-Huson University College, Al-Balqa Applied University, Al-Salt 19117, Jordan; 3 Department of Animal Production, Faculty of Agriculture, Jordan University of Science and Technology, P.O. Box 3030, Irbid 22110, Jordan; bobeidat@just.edu.jo; 4 Department of Oncology, King Hussein Cancer Center, Amman 11941, Jordan; fa.11194@khcc.jo; 5 College of Health Sciences, QU Health, Qatar University, Doha P.O. Box 2713, Qatar; hbawadi@qu.edu.qa

**Keywords:** cancer, nutrition, mental health, dietary intake, treatment

## Abstract

Chemotherapy can negatively affect cancer patients’ eating patterns, psychological status, body composition, and quality of life. In this study, we aimed to investigate the correlations between dietary intake/care and the psychological status of cancer patients treated with chemotherapy. An observational study was conducted on 75 participants during their first cycle of chemotherapy treatment, and they were followed up for three cycles. Each participant completed a reliable validated questionnaire, psychological questionnaire, quality of life questionnaire, and three-day food records. Dietary intake was considered adequate if there was an adherence of participants to dietary recommendations offered by the dietitian and was confirmed by ESHA analysis software (version 10.6.3). Seventy-five percent of participants had inadequate nutrition intake. All anthropometric measurements decreased after 2 months of chemotherapy regardless of patients’ dietary intake. Approximately half of the participants reported depression and anxiety. There were significant differences between all nutrient intake levels when compared to the recommended dietary allowance except for fat, unsaturated fatty acids, and iron. Also, there were associations between nutritional intake and life quality and depression. In conclusion, poor dietary intake was associated with depression and insufficient macro- and micronutrient intake. Emotional and nutritional support from healthcare providers and family are highly necessary.

## 1. Introduction

Cancer is a major public health problem worldwide. It is an important cause of morbidity and mortality in both low and economically developed countries [1]. Cancer is the leading major cause of death in the 21st century. In 2022, 1,918,030 new cancer cases and 609,360 cancer deaths were projected to occur in the United States [2]. Worldwide, an estimated 19.3 million new cancer cases and almost 10.0 million cancer deaths occurred in 2020 [3]. Chemotherapy—a common treatment for various types of cancer—is associated with psychological, nutritional, and health complications that affect the quality of patients’ lives. The side effects of chemotherapy may include loss of taste, mucositis, xerostomia, dysphagia, nausea, swallowing difficulties, vomiting, diarrhea, and toxicities [4]. With time, these side effects lead to a reduction in food intake, starvation, cachexia, malnutrition, weight loss, loss of lean body mass, hair loss, muscle wasting, alteration in eating behaviors, changes in the body metabolism, protein calorie malnutrition, stress, anxiety, and depression [3,5]. 

Nutrition plays an important role in cancer treatment prognosis [6,7]. Therefore, cancer patients must always be assessed anthropometrically, nutritionally, and biochemically. Meanwhile poor nutritional status may worsen complications [8,9]. Generally, cancer affects the psychological status of patients who usually suffer from anxiety, depression, stress, and mood disorders [10]. Studies reported that patients who received practical and emotional family support had decreased levels of stress and depression [11]. 

Malnutrition is a common feature in cancer patients and is the consequence of medical and surgical anticancer treatments such as chemotherapy. Malnutrition negatively impacts the patient’s quality of life, and it has been estimated that up to 10–20% of cancer patients die due to complications of malnutrition rather than the tumor itself. Therefore, nutrition plays a vital role in cancer care. Nutrition care should be taken into account from the time of cancer diagnosis, within a diagnostic and therapeutic pathway, and should be working simultaneously with anticancer treatments [12]. Malnutrition associated with cancer differs from starvation-related malnutrition as it results from a combination of anorexia and metabolic dysregulation, caused by the tumor itself or by its treatment. Cancer-associated malnutrition can lead to a multifactorial syndrome known as cachexia, characterized by the severe, involuntary loss of skeletal muscle mass, with or without the loss of fat mass, an increased systemic inflammatory response [13], and increased protein catabolism [14]. Malnutrition, risk of malnutrition, and depressive symptoms often occur among patients, and the relationship between them was statistically significant. Thus, the quality of life of cancer patients could be improved by providing adequate nutrition and psychological support [15]. The functional consequences of malnutrition not only cause physical changes but also psychological changes such as depression, anxiety, irritability, apathy, poor sleep patterns, and loss of concentration [16]. Previous studies have shown that providing adequate nutrition to cancer patients can help prevent malnutrition and its consequences, as well as improve psychological status by decreasing stress and depression levels. The study hypothesis indicates that body composition, biochemical tests, and the psychological health of cancer patients could be improved by providing adequate nutrition. Therefore, the objective of this study was to assess the association between adequate nutrient intake and nutritional care on the psychological and physical wellbeing of cancer patients receiving chemotherapy treatment.

## 2. Materials and Methods

### 2.1. Study Design, Participants, and Questionnaire 

This study is a cross-sectional investigation conducted at King Hussein Cancer Center (KHCC). The study was performed in accordance with the Declaration of Helsinki and has been approved by the KHCC ethics committee (Approval Code: 18 KHCC 45, Approval Date: 11 December 2017), Deanship of Research—Jordan University of Science and Technology (459/2019), and the Institutional Review Board (IRB) of King Abdullah University Hospital. The sample size was estimated to be 60 patients within a margin error of 5% and a confidence level of 99% was estimated as 55 subjects, but for a higher power, the sample size was expanded to 75 participants from the Oncology Department at King Hussein Cancer Center (KHCC) in Amman/Jordan. Patients aged 18–60 years old, females and males, who came to their first cycle chemotherapy treatment were included in the study. All participants filled out a reliable and validated questionnaire after signing a consent form that confirmed their approval to participate in this study. 

### 2.2. Psychological Status

The psychological status of the participants, mainly depression and anxiety, was assessed using a validated questionnaire composed of a scale adopted from the Hospital Anxiety and Depression Scale (HADS) [17]. The scale was given to each participant to be completed after the first chemotherapy dose, and the scores were calculated based on the answers.

### 2.3. Quality of Life Assessment 

To determine the participants’ quality of life, each participant was asked to complete a questionnaire adopted from EORTC QLQ-C30 [18]. This questionnaire assesses three scales: functional, symptoms, and health status. Questions included (daily activities, pain, tiered, appetite, breath, vomiting, constipation, diarrhea, nausea, sleep, tense, worry, depression, overall health, and quality of life). The questionnaire was completed after the first dose of chemotherapy, and the scores were calculated based on the answers.

### 2.4. Anthropometric Measurements 

**Figure 1:**
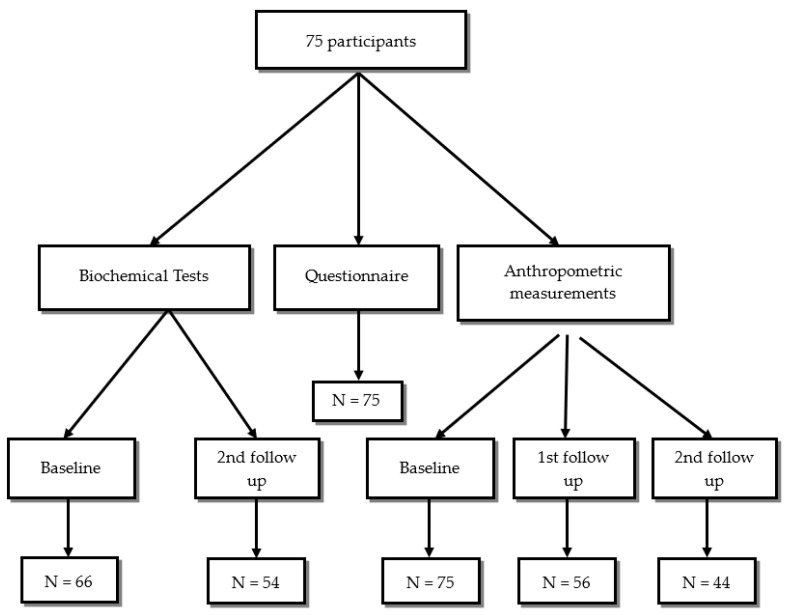
Inclusion and follow-up for study participants.

Body weight in kilograms, fat mass, muscle mass, minerals, body fluids in litters, body mass index (BMI) kg/m^2^, waist/hip ratio, and BMR (Basal Metabolic Rate) were measured using the body composition analyzer (Inbody 270, Seoul, Republic of Korea). All preparatory steps for the InBody Test were conducted to ensure consistent testing conditions and the most accurate results. The guidelines include maintaining a normal fluid intake the day before, standing upright for at least 5 min, removing any socks or pantyhose, and removing all heavy accessories like jewelry, watches, and jackets. Each measurement was taken 3 times, at the first visit (baseline), one month later (first follow-up), and after two months (second follow-up). The baseline anthropometric measurements were taken from all subjects (*n* = 75), but on the first follow-up, data were collected from 56 participants only, while data from 44 participants only were obtained on the second follow-up (Figure 1).

### 2.5. Dietary Intake

The participants’ dietary intake was assessed using the three-day food record which was given to each participant after the first chemotherapy dose. Collected data were analyzed by ESHA (version 10.6.3). The adherence of participants and their family members to dietary recommendations is expected to influence macro- and micronutrient intake. Data were analyzed and compared with recommended dietary intake (RDA). Participants were categorized into two groups according to their dietary intake, adequate if their dietary intake met RDA and inadequate if their dietary intake was below RDA [19,20]. Nutrition care was considered available if there was an adherence of participants and their family members to the dietary recommendation offered by the institutions’ dietitian. 

### 2.6. Biochemical Analysis

The routine biochemical tests in KHCC included complete blood count (CBC), kidney function tests, and liver function tests, and these were collected at baseline, first follow-up, and on the second follow-up. In our study, 66 of the participants had baseline biochemical tests, while 54 participants had their second follow-up visit. The routine biochemical tests were taken for the participants twice based on the physicians’ request. The number of participants at baseline, first follow-up, and second follow-up is described in Figure 1. Statistical analysis was run on the 54 participants who have completed both initial (baseline) and after 3 months of chemotherapy tests. 

### 2.7. Statistical Analyses

Data were collected, encoded, and analyzed using the SPSS statistical package version 22 [21]. Descriptive statistics were performed using means and standard deviations (SDs) to describe continuous variables and frequencies and percentages to describe the categorial variables. All continuous variables were examined for normal distribution using the nonparametric Kolmogorov–Smirnov test. Moreover, the paired-sample *t* test was used to test the differences between the means of the actual dietary intake and the recommended dietary intake. The Chi-square test was used to test the differences between the frequencies of dietary intake, quality of life, and psychological status. When variables followed significantly skewed distribution, medians were compared using the Mann–Whitney U test for the independent samples and the Wilcoxon signed rank test for paired-samples. All associations and differences were considered significant at a *p*-value of ≤0.05. 

## 3. Results

### 3.1. Socio-Demographic, Cancer Types, and Cancer-Related Characteristics

Most participants were females (74.7%), and 46.7% of them were aged between (31 and 45 years old). Approximately 46.7% had a low educational level, while 5.3% had a high educational level (Master or Ph.D.), and the majority (96%) had health insurance. Around three-quarters of participants were married (76%), and about 54.7% had a family history of cancer. Forty percent of the study participants were diagnosed with breast cancer, and the rest were diagnosed with other cancer types, such as: colon (9.3%), lymphoma (22.2%), lung (12%), and liver (10.7%). 

All participants (100%) were taking chemotherapy, and they were on the first cycle of their treatment. Forty percent of the participants were diagnosed by ultrasound and CT scan, and 20% were diagnosed by PET scan. Half of the participants showed abnormal depression and anxiety status (46.7% and 53.3%), respectively. The results of the QLS revealed that 69.3% had bad health status, 36% had bad symptoms, and 44% had bad functional status. A total of 74.7% of the participants did not meet their macro- and micronutrients recommended intake, and they were considered as “not taking adequate nutritional care”, while 25.3% of them only received adequate nutritional care, which means they met the requirements of both macro- and micronutrients intake. 

### 3.2. Anthropometric Measurements before the First Cycle of Chemotherapy and after the Third Cycle

Table 1 shows the anthropometric measurements of the study subjects at baseline and after 3 months. All the anthropometric measurements and body composition variables significantly (*p* < 0.05) decreased after three cycles of chemotherapy treatments, except for the waist/hip ratio (WHR), which showed no significant difference (*p* > 0.05). The correlation between the biochemical tests of all the participants and nutritional care is shown in Section 3.3.

### 3.3. Correlation between Adequate and Inadequate Dietary Intake with Psychological Status, Quality of Life, Anthropometric Measurements, and Biochemical Tests

More than half of the participants (53.6%) who had an inadequate dietary intake had depression (*p* = 0.000), while 26.3% of those who had an adequate dietary intake were suffering from depression. On the other hand, 58.9% of those who had an inadequate nutrients intake had anxiety, but this does not correlate significantly with dietary intake. There were significant associations between both functional and healthcare scale and dietary intake (*p* = 0.00), while no significant (*p* > 0.05) association was observed between dietary intake and symptoms scale (Table 2). More than half of the participants (53.6%) who had an inadequate nutrients intake had depression (*p* = 0.000), while 26.3% of those who had an adequate nutrients intake were suffering from depression. On the other hand, 58.9% of those who had inadequate nutrition care had anxiety, but this does not correlate significantly with nutrients adequacy. Table 2 also shows the correlation between nutritional care status (dietary intake) and anthropometric measurements. There was no significant (*p* > 0.05) correlation between the nutritional care adequacy and body fat percentage, waist/hip ratio, BMI, body water, minerals, fat mass, and muscle mass.

There were no significant (*p* > 0.05) associations between biochemical tests and nutritional care adequacy as shown in Table 3.

### 3.4. Dietary Intake (Macro- and Micronutrients) of Study Sample Compared to Recommended Dietary Intake for Cancer Patients

Table 4 and Table 5 showed that the mean of the participants’ macro- and micronutrients intake was significantly different for all macro- and micronutrient intake when compared to RDA for cancer patients, except for fat, saturated fat, and iron.

## 4. Discussion

The results of this study partially supported our hypothesis, which is the correlation between the dietary intake and psychological status of chemotherapy-treated cancer patients. Body composition, biochemical tests, and anthropometric measurements were not affected by dietary intake adequacy. Nho et al. [22] found that there was no significant difference in the prevalence of malnutrition and inadequate dietary intake according to cancer type, which agreed with our findings. About half of the participants in this study had a family history of cancer. After three months of chemotherapy treatment, there was a substantial decrease in anthropometric and body composition indices. There was also a strong correlation between good nutrition status, access to quality healthcare, and functional quality of life (QoL) ratings. This association was directly and positively reflected in the participants’ biochemical results.

On the other hand, inadequate nutrient intake was significantly associated with borderline and abnormal depression. Nho et al. [22] found no significant difference in the prevalence of malnutrition and inadequate nutritional care according to cancer type, which is consistent with our findings. In this study, most participants were not consuming enough fruits, vegetables, and water, and not following a healthy diet during chemotherapy treatment. Interestingly, besides the inadequate family care and attention to their dietary intake and quality of food items consumed by their ill family member, chemotherapy complications may significantly affect cancer patients’ intake by causing loss of taste, vomiting, diarrhea, constipation, and nausea [23]. Cancer patients reported sleep disturbance associated with depression, as mentioned also by Clevenger et al. [24], who indicated that sleeping disturbance is common in cancer patients and is associated with depression; if depression increases, sleep disturbance increases (*p* < 0.04). 

Patients under cancer treatment suffer from stress because of a lack of motivation or fatigue, which is a trigger for anxiety and depression [25]. Nikbakhsh et al. [25] revealed that about half of their study’s participants had mild and symptomatic anxiety and similar results for depression, which is consistent with our results. A study of head and neck cancer, reported by Rieke et al. [26], indicated that 18.5% of participants were diagnosed with depression. Similarly, Ma et al. [27] found that there was a significant positive correlation between poor nutrition status and level of depression and anxiety. There may be a connection between cancer-related anorexia and depression since the condition, which is characterized by a substantial loss of appetite, appears to be linked to serotonin impairment, a neurotransmitter that is a biomarker for depression [27]. Nho et al. [22] reported that loss of appetite may be a common feature of depression and anxiety, leading directly to malnutrition. Marital status, which indicates the family support system, was not related to malnutrition. The authors also confirmed that family support or social support is reported as a significant factor correlated with nutritional risks [22]. The high incidence of depression and anxiety in our study may be due to poor family and/or friends support, disease, and treatment complications. Also, a high percentage of the patients in our study reported poor quality of life based on the health, functional, and symptoms status scale, which may affect their mental and psychological health significantly. 

Other studies’ findings were not in agreement with our findings, such as Kiss et al. [28], who reported that there was no association between patients’ nutrition and quality of life. Vergara et al. [29] found that nutrition-related symptoms, or gastro-intestinal symptoms, negatively impact patients’ quality of life, which did not come in concordance with our results. A study of Mardas et al. [30] showed that there was no statistically significant correlation between symptom status evaluation of the quality of life scale and dietary intake, which is similar to our results. Symptom status is more likely to be correlated with treatment type and dose rather than dietary intake and nutritional/family care.

As expected, BMI and weight were reduced at the second follow-up compared to the baseline anthropometric assessment. This could be due to chemotherapy complications, depression, and lack of nutritional intake. This finding is in line with Soilheim et al.’s findings [31], who reported that patients treated with chemotherapy suffer from low appetite and weight loss. Abnormal metabolism, catabolic processes, treatment complications, and reduced caloric intake due to underlying malignancy are the main causes of muscle loss within cancer patients [29,32]. Stene et al. [32] also found that there was a reduction in mean muscles mass among patients receiving chemotherapy. Body water amount also decreased, mainly because of taste change as a treatment complication and the loss of fat free mass. Body fat and protein percentage decreased significantly due to malnutrition and chemotherapy complications, which supported our finding regarding body composition reduction [32]. Inadequate nutrition negatively impacts treatment prognosis by increasing the risk of infection, increasing treatment toxicity, delaying wound healing, prolonging hospital stay, and increasing health costs [29].

The relationships between cancer and pro-inflammatory cytokines often lead to nutritional deterioration and poor quality of life; cytokines influence the balance of anorexigenic circuits and orexigenic, that predispose to cancer anorexia–cachexia, which involve the interplay of mediators, including hormones like neuropeptides, leptin, cytokines (e.g., interferon, interleukin 6 (IL-6), tumor necrosis factor alpha [TNF-α], interleukin 1 (IL-1)), and differentiation factor [13]. IL-6, IL-1, and TNF-α decrease the intake of food, increase glucose oxidation, gluconeogenesis, the synthesis of acute phase reactive proteins, increase the hepatic synthesis of fatty acids, and the resting energy expenditure, but they decrease the uptake of fatty acids. Cytokines also affect metabolism by altering glucagon, insulin, and corticosterone levels. IL-6 and TNF-α were also believed to be associated with muscle wasting [29]. 

Malihi et al. [33] found that macronutrient intake, such as daily energy intake, carbohydrates, fat, and protein, significantly declined after the first induction of chemotherapy, which supports our results in assessing the dietary intake after the first chemotherapy cycle. Nonetheless, a lack of macronutrient and micronutrient intake may be related to appetite loss in cancer patients who receive chemotherapy [22].

## 5. Conclusions

In our study, cancer patients on chemotherapy treatment mostly did not have an adequate dietary intake, and they were more vulnerable to cachexia and malnutrition. Inadequate nutritional status plays a major role in increasing patients’ depression/anxiety scales. Nutritional care and family support is highly recommended for patients on chemotherapy treatments to improve their functional health status and life quality overall.

## Figures and Tables

**Table 1:** Anthropometric measurements at baseline and after 3 months of chemotherapy in cancer patients (*n* = 44).

	Initial Mean (SD)	Final Mean (SD)	*p*-Value
Weight (kg)	76.8 (18.2)	66.8 (13.9)	0.00
Height (cm)	163.2 (8.2)	163.2 (8.2)	ND *
Muscles mass (kg)	26.7 (6.7)	23.4 (5.7)	0.00
Fat mass (kg)	28.2 (11.8)	23.2 (8.2)	0.00
Minerals (kg)	3.4 (0.7)	3.0 (0.7)	0.00
Body water (L)	35.7 (8.1)	32.1 (7.3)	0.00
Waist/hip ratio	0.9 (0.1)	0.9 (0.1)	0.48
Fat%	36.1 (9.0)	32.7 (9.2)	0.01
BMI ** (kg/m^2^)	28.9 (6.5)	25.2 (4.8)	0.00
Basel metabolic rate (kcal)	1419.7 (238.8)	1293.3 (217.9)	0.00
Protein (kg)	9.5 (2.2)	8.5 (2.0)	0.00
Visceral fat (level)	12.7 (4.6)	10.4 (4.4)	0.00

* ND: not determine; BMI **: Body mass index: normal weight (18.6–24.9 kg/m^2^), overweight (25–29.9 kg/m^2^), obese grade I (30–34.9 kg/m^2^), obese grade II (35–35.9 kg/m^2^), and obese grade III (≥40 kg/m^2^).

**Table 2:** Correlation between adequate/inadequate dietary intake and psychological status, quality of life, and body composition in cancer patients undergoing chemotherapy (*n* = 75).

Variables	Dietary Intake		*p*-Value
	Inadequate *n* (%)	Adequate *n* (%)	
Psychological status			
Depression			0.00
Normal	3 (5.4)	7 (36.8)	
Borderline abnormal	23 (41)	7(36.8)	
Abnormal	30 (53.6)	5 (26.3)	
Anxiety			0.25
Normal	8 (14.3)	4 (21.1)	
Borderline abnormal	15 (26.8)	8 (42.1)	
Abnormal	33 (58.9)	7 (36.8)	
Quality of life			
Healthcare scale			0.00
Bad	44 (78.6)	8 (42.1)	
Good	12 (21.4)	11 (57.9)	
Functional scale			0.01
Bad	13 (68.4)	20 (35.7)	
Good	6 (31.6)	36 (64.3)	
Symptom’s scale			0.64
Bad	21 (37.5)	6 (31.6)	
Good	35 (62.5)	13 (68.4)	
Anthropometric measurements and body composition (Mean ± SD)			
Fat (%)	32.6 ± 9.9	32.6 ± 7.5	0.74
Waist/hip ratio	0.9 ± 0.1	0.9 ± 0.1	0.66
BMI (kg/m^2^)	25.5 ± 5.3	24.6 ± 3.6	0.23
Body water (L)	32.3 ± 7.6	31.7 ± 6.6	0.81
Minerals (kg)	2.9 ± 0.7	3 ± 0.5	0.93
Fat mass (kg)	23.4 ± 8.5	22.9 ± 7.6	0.46
Muscle mass (kg)	23.4 ± 5.8	23.4 ± 5.4	0.64

Chi-square test was used to test the differences between the frequencies of dietary intake, quality of life, and psychological status. All associations and differences were considered significant at a *p*-value of ≤0.05.

**Table 3:** Correlation between adequate/inadequate dietary intake and biochemical tests in cancer patients undergoing chemotherapy (*n* = 54).

Biochemical Tests	Dietary Intake				*p*-Value
	Inadequate		Adequate		
	Median	Range	Median	Range	
WBC	5.7	0.1–36.5	6.2	1.3–11.4	0.83
RBC	4.12	1.09–5.29	3.9	3.3–5.89	0.86
MCV	86.3	73.8–97.6	81.2	35.8–99.8	0.42
MCH	29.1	24.2–33.6	30	15.5–33.1	0.81
MCHC	34	32.4–36	33.8	30.5–35.2	0.18
RDW	16.8	12.2–22.6	18.4	13.7–27.2	0.11
Platelet count	271	20–566	248	129–455	0.91
MPV	8.5	4.8–11.4	8.8	7.9–17.5	0.16
Lymphocyte	23.8	3.2–54.3	26.6	8.5–48.7	0.98
Monocytes	9.7	0.9–41.8	9.3	1.5–42.4	0.97
Eosinophile	1.3	0–9	0.4	0–6.9	0.25
Basophile	0.7	0–1.5	0.7	0.2–1.4	0.82
HB	12	7–15.9	11.2	8.1–15.1	0.17
Neutrophils	62.4	19–95	64.5	5–87	0.86
PCV	35.9	21–44.3	33.9	26.7–46.3	0.26
Serum creatinine level	0.61	0.1–1.34	0.6	0.44–1.04	0.37
Sodium serum level	140	133–143	140	136–142	0.79
Potassium	4.56	3.74–5.52	4.4	3.63–5.2	0.51
Chloride serum level	101.7	91.7–106.4	101.4	94.7–103.7	0.79
Uric acid serum	4.78	1.6–7.7	4.46	3.25–6.48	0.30
Albumin serum level	4.3	2.56–5	4.355	3.64–5.13	0.44
Bilirubin serum level	0.32	0.15–1.41	0.465	0.18–24	0.10
Alkaline phosphatase	83	40–924	68	50–94	0.08
AST	18.8	0–129	17.4	0–43	0.57
ALT	16.8	0–109	13	0–47	0.78
Bilirubin direct	0.11	0–0.37	0.135	0–0.27	0.57
Total protein serum	6.74	0–8.6	6.85	0–7.64	0.94
Urea serum level	21	0–48	17.5	0–38	0.55

Mann–Whitney U Test.

**Table 4:** Daily macronutrient intake in cancer patients taking chemotherapy compared to RDA specific for cancer patients (*n* = 74).

	Mean ± SD	RDA *	*p*-Value
Calories (kcal)	1452 ± 1061.1	30 kcal/kg/day	0.00
Protein (g)	57 ± 52.0	2 g/kg	0.00
Carbohydrate (g)	173 ± 127.2	100 g/day	0.00
Fiber (g)	15 ± 21.1	30 g/day	0.00
Fat (g)	61 ± 66.2	55 g/day	0.40
Saturated fat %	7 ± 3.5	7	0.09
Unsaturated fat%	12 ± 10.8	15	0.02
Omega 3 and omega 6 (g)	8 ± 26.7	250 mg	0.01
Protein %	17 ± 7.1	30	0.00
Carbohydrate %	49 ± 12.8	45	0.00
Fat %	34 ± 13.9	25	0.00

Paired-sample *t* test was used to test the differences between the means of the actual dietary intake and the recommended dietary intake. * RDA: recommended dietary allowance for cancer patients. All associations and differences were considered significant at a *p*-value of ≤0.05.

**Table 5:** Daily micronutrient intake of cancer patients taking chemotherapy compared to RDA specific to cancer patients (*n* = 74).

Vitamins and Minerals	Mean ± SD	Recommended Dietary Allowance *	*p*-Value
Vitamin B_1_ (mg)	0.5 ± 0.4	1.5	0.00
Vitamin B_2_ (mg)	0.6 ± 0.5	1.7	0.00
Vitamin A (IU)	3059.1 ± 3194.1	40,000	0.00
Vitamin B_12_ (mcg)	1.1 ± 2.7	6	0.00
Folate (mcg)	134.0 ± 123.7	400	0.00
Vitamin E (mg)	2.8 ± 5.3	15	0.00
Vitamin D (IU)	30.2 ± 52.1	400	0.00
Vitamin B_3_ (mg)	7.1 ± 6.0	20	0.00
Vitamin B_6_ (mg)	0.5 ± 0.4	100	0.00
Vitamin C (mg)	33.2 ± 25.7	2000	0.00
Sodium (mg)	3426.5 ± 3400.8	1500	0.00
Magnesium (mg)	77.9 ± 52.4	370	0.00
Selenium (mcg)	18.3 ± 18.8	55	0.00
Zinc (mg)	2.8 ± 2.4	90	0.00
Iron(mg)	9.1 ± 9.5	11	0.09

Paired-sample *t* test was used to test the differences between the means of the actual dietary intake and the recommended dietary intake. * RDA: recommended dietary allowance for cancer patients. All associations and differences were considered significant at *p*-value of ≤0.05.

## Data Availability

The data will be provided upon request from the corresponding authors.
